# Supplementary material for: NaCl Modifies Biochemical Traits in Bacterial Endophytes Isolated from Halophytes: Towards Salinity Stress Mitigation Using Consortia
Source: Plants (Basel). 2024 Jun 12;13(12):1626. doi: 10.3390/plants13121626 (PMC11207235; doi:10.3390/plants13121626)
Supplement: Supplementary file 1 [file plants-13-01626-s001.zip › plants-2948840-supplementary.pdf]

Table S1: Electrical conductivity (EC) of rhizosphere soil and molecular identification of endophyte bacteria isolated from six halophytes.

Halophyte	EC (dS m^{-1})	Bacterial code	Molecular identification	
			Sequence homology (% identity)	NCBI accession number
D. spicata	16	1	99	SAMN40421649
D. spicata	12	2	99	SAMN40421650
K. scoparia	7	3	99	SAMN40421651
S. torreyana	12	4	99	SAMN40421652
C. dactylon	6	5	99	SAMN40421653
D. spicata	8	6	99	SAMN40421654
K. scoparia	5	7	99	SAMN40421655
K. scoparia	8	8	99	SAMN40421656
E. obtusiflora	9	9	99	SAMN40421657
S. torreyana	7	10	99	SAMN40421658
B. salicifolia	6	11	99	SAMN40421659

Table S2: Qualitative nitrogenase determination and exopolysaccharides produced by halophilic endophytic bateria isolated from halophytes.

Isolate	Nitrogenase enzyme		Exopolysaccharide	
	NaCl 0M	NaCl 2.5M	NaCl 0M	NaCl 2.5M
1	-	-	+	+
2	-	-	-	-
3	+	+	-	-
4	+	+++	+	+
5	-	-	+	+
6	-	-	+	+
7	+	+	-	-
8	+++	++	-	-
9	++	+++	-	-
10	-	+++	-	-
11	-	-	-	-

-,+,++,+++ denotes: none, low, moderate and high nitrogenase enzyme response, respectively in 11 endophytic bacteria. Exopolysaccharide production: (+) with or (-) without production. n=3.

Table S3: Growth of 11 bacteria in the nutrient solubilization tests.

	P-$Ca_3(PO_4)_2$		P-($C_6H_{18}O_{24}P_6$)		Feldspar K		ZnO		MnO	
Isolate	0 M NaCl	2.5 M NaCl	0 M NaCl	2.5 M NaCl	0 M NaCl	2.5 M NaCl	0 M NaCl	2.5 M NaCl	0 M NaCl	2.5 M NaCl
1	0.50±0.02A	0.53±0.03a	0.61±0.01A	0.61±0.02a	0.55±0.02A	0.58±0.02a	0.52±0.02A	0.53±0.01a	0.60±0.02A	0.65±0.02a
2	0.56±0.02A	0.56±0.02a	0.59±0.01A	0.60±0.02a	0.58±0.02A	0.58±0.02a	0.50±0.01A	0.55±0.01a	0.62±0.02A	0.64±0.01a
3	0.52±0.02A	0.55±0.03a	0.59±0.02A	0.61±0.01a	0.56±0.01A	0.56±0.01a	0.50±0.01A	0.51±0.02a	0.58±0.02A	0.62±0.02a
4	0.52±0.03A	0.57±0.02a	0.57±0.02A	0.62±0.02a	0.58±0.03A	0.61±0.01a	0.51±0.01A	0.53±0.01a	0.63±0.01A	0.66±0.01a
5	0.52±0.02A	0.54±0.02a	0.56±0.02A	0.61±0.02a	0.52±0.02A	0.55±0.03a	0.51±0.01A	0.55±0.02a	0.61±0.03A	0.63±0.03a
6	0.53±0.02A	0.54±0.02a	0.60±0.02A	0.61±0.01a	0.55±0.02A	0.56±0.03a	0.51±0.01A	0.54±0.03a	0.61±0.01A	0.63±0.03a
7	0.51±0.01A	0.56±0.01a	0.60±0.01A	0.63±0.01a	0.58±0.01A	0.60±0.02a	0.53±0.01A	0.54±0.02a	0.64±0.02A	0.64±0.02a
8	0.50±0.02A	0.56±0.03a	0.60±0.01A	0.59±0.01a	0.54±0.01A	0.61±0.02a	0.50±0.01A	0.58±0.01a	0.60±0.02A	0.66±0.02a
9	0.53±0.03A	0.54±0.02a	0.62±0.03A	0.65±0.03a	0.56±0.02A	0.58±0.01a	0.52±0.01A	0.55±0.03a	0.62±0.02A	0.63±0.01a
10	0.54±0.02A	0.55±0.01a	0.59±0.02A	0.64±0.02a	0.52±0.02A	0.56±0.01a	0.53±0.02A	0.53±0.02a	0.66±0.02A	0.60±0.02a
11	0.51±0.01A	0.56±0.01a	0.59±0.01A	0.63±0.01a	0.56±0.02A	0.58±0.02a	0.52±0.01A	0.52±0.03a	0.61±0.01A	0.62±0.02a

Absorbance was used as indirect measure of bacterial growth. Similar capital letters show no statistical difference when comparing among bacterial growth at 0 M NaCl. Similar lowercase letter show no statistical difference when comparing among bacterial growth at 2.5 M NaCl.

Table S4: Compatibility among halophilic endophytic bacteria isolated from halophytes.

Isolate/Isolate	1	2	3	4	5	6	7	8	9	10	11
1		+	+	+	+	+	+	+	+	+	+
2	+		+	+	+	+	+	+	+	+	+
3	+	+		-	+	+	-	+	+	-	+
4	+	+	-		+	+	+	+	+	+	+
5	+	+	+	+		-	+	+	+	+	+
6	+	+	+	+	-		+	+	+	-	+
7	+	+	-	+	+	+		-	+	+	+
8	+	+	+	+	+	+	+		+	+	+
9	+	+	+	+	+	+	+	+		+	+
10	+	+	-	+	+	+	+	+	+		+
11	+	+	+	+	+	+	+	+	+	+	

+, - show compatibility or incompatibility among the 11 bacterial isolates, respectively.

Table S5: Eigenvalues, variance, and stronger variables from principal component analysis.

Biochemical trait	Eigenvalues	Variance (%)
Nitrogenase 2.5 M*	3.86	31.33
Phosphate from organic source	3.24	19.64
Fitase 2.5 M	2.86	16.45
Fructuose 2.5 M	2.18	12.90
Potassium 2.5 M	1.18	10.56
Phosphate-from organic source 2.5 M	0.86	8.94
Zinc 2.5 M	0.50	7.52
Zinc	0.38	6.90
Siderophores 2.5 M	0.28	5.63
Siderophores	0.21	4.94
Indol Acetic Acid 2.5 M	0.19	4.63
Potassium	0.17	4.29
Citric acid 2.5 M	0.15	3.29
Citric acid	0.12	2.91
Phosphate from inorganic source 2.5 M	0.10	2.58
Phosphate from inorganic source	0.8	2.37
Indol Acetic Acid	0.4	2.27
Acid phosphatase	0.2	1.85
Exopolysaccharides 2.5 M	0.08	1.07
Exopolysaccharides	0.07	0.95
Nitrogenase	0.05	0.65

* Denote response in presence of NaCl

Table S6: 16S gen amplification process by PCR to molecular identification of halophilic endophytic bacteria.

Stage	Time (seconds)	Temperature (°C)	Cycles
Initial denaturalization	300	94	1
Denaturalization	30	94	35
Alignment	30	48	35
Extension	150	72	35
Final extension	300	72	1
Maintenance		10	1

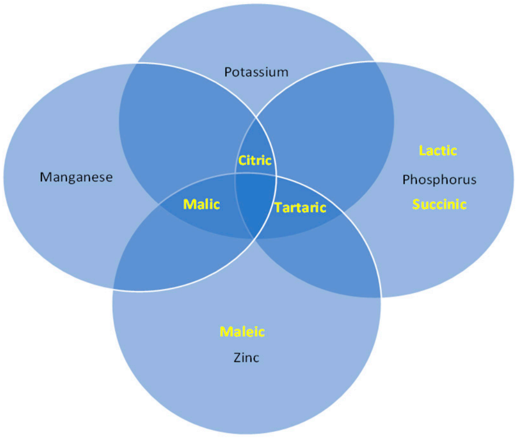

Figure S1: Venn´s diagram presenting the organic acids secreted in each solubilization ion and common among solubilization tests.

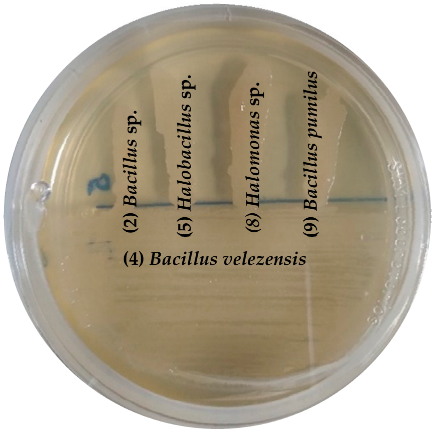

Figure S2. Bacterial compatibility of one selected consortium with beneficial properties such as plant growth promotion, nutrient solubilization, and enzyme production.

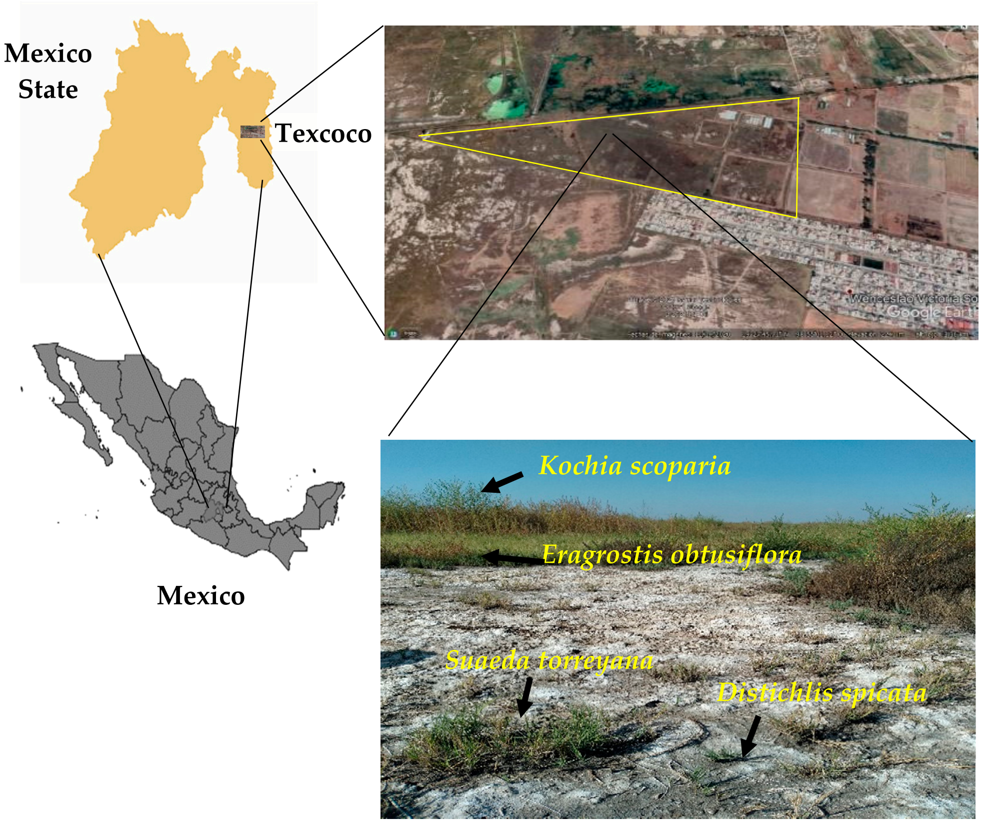

Figure S3: Location of sampling site to the isolation of endophytic bacteria from roots of six halophytes.
